# Chromosome centromere copy number amplification associated with exceptional response in HER2-positive metastatic breast cancer patients

**DOI:** 10.1038/s41388-025-03667-8

**Published:** 2025-12-20

**Authors:** Charlotte Andrieu, Jojanneke Stoof, Dalal AlSultan, Laura Ivers, Jose Javier Berenguer Pina, Darko Skrobo, Jo Ballot, Debbie O’Reilly, Denis M. Collins, Alex J. Eustace, Cecily Quinn, Janice M. Walshe, Giuseppe Gullo, Naomi Walsh, John Crown

**Affiliations:** 1 Life Sciences Institute, School of Biotechnology, Dublin City University (DCU), Dublin, Ireland; 2 Department of Medical Oncology, St Vincent’s University Hospital, Dublin, Ireland; 3 Department of Histopathology, Galway University Hospital, Galway, Ireland; 4 Department of Pathology, St Vincent’s University Hospital, Dublin, Ireland

**Keywords:** Prognostic markers, Cancer genomics, Breast cancer

## Abstract

Metastatic breast cancer (MBC) is generally an incurable neoplasm. A small cohort of patients with HER2-positive MBC, however, achieve such prolonged remission without relapse following anti-HER2 therapy and chemotherapy, that it is speculated they might be cured. The genomes of these patients might provide insights into the underlying mechanisms for their successful treatment. Here, a total of 243 HER2-positive patients diagnosed with MBC between 2000 and 2015 were studied. Of these, 29 patients were identified as exceptional responders (ExR) with an overall survival (OS) > 60 months and no evidence of relapse, 54 patients with an OS > 60 months but who relapsed or developed progressive disease were defined as exceptional survivors (ExS), and 160 patients with an OS < 60 months were identified as short-term responders (STR). Whole-Genome Sequencing and centromere copy number (CCN) analysis was performed on 27 patients (12 ExR; 4 ExS; 11 STR). A significant amplification was observed in the centromeric regions of ExR, exhibiting higher CCN compared to the ExS and STR. Digital PCR validation of chromosome 4 centromere region D4Z1 copy number was not associated with ExR OS. Our results suggest that the amplification of centromere regions are associated with very prolonged remission and survival in patients with HER2-positive MBC.

## Introduction

Despite advances in chemotherapy and endocrine therapy, metastatic breast cancer remains a generally incurable disease. An exception may be found within the minority of patients whose cancers harbor an alteration of the HER2 gene (usually an amplification) and/or an over-expression in the HER2 protein. For these patients, whose cancer is more intrinsically aggressive, the addition of the monoclonal antibodies trastuzumab and subsequently pertuzumab to conventional chemotherapy have resulted in improved survival. For patients whose cancer has relapsed following, or was resistant to these, now-conventional chemotherapy and antibody treatments, the use of HER2 targeting small molecule tyrosine kinase inhibitors and antibody-drug conjugates in the second and subsequent lines of therapy can produce further improvements [1–5]. We and others have reported the intriguing observation that a meaningful minority of patients with HER2 altered disease who were treated with chemotherapy and trastuzumab achieved unusually long continuous remission [6–12]. We speculated that some of these patients might be cured. Exceptional response in solid cancers is a rare event, and the criteria are specific to the type of cancer, its stage and treatment regimen [13]. The National Cancer Institute (NCI) defined an exceptional response as a complete or partial response observed in less than 10% of similarly treated patients or a duration of response lasting three times the published median or longer [14]. However, this terminology is best suited to novel agents as low response rates would not tend to be standard of care. Due to the lack of consensus among researchers regarding the definition of exceptional patients, and our extensive progression-free survival and overall survival follow-up of 20+ years for HER2-positive MBC patients treated with the established therapy trastuzumab plus taxane, we termed exceptional responders (ExR) as HER2-positive MBC who achieve an overall survival greater than 5 years (60 months) with no evidence of relapse.

As part of an ongoing project to characterize the clinical, genomic, and immunological characteristics of these “exceptional responders”, we previously performed a small pilot genomic study using whole exome sequencing to assess the possible impact of copy number aberrations on the likelihood of achieving durable remissions. We observed a numerically lower copy number (CN) burden in patients who achieved prolonged remission compared to non-responders [11], a finding which has been confirmed by others [15]; however no studies to date have assessed the clinical impact of the whole genomic landscape including the non-coding region in HER2-positive MBC, and the possible influence on long-term outcome. In this study, we sought to investigate the association of chromosomal abnormalities across the full genome with prognosis and treatment response by comparing CNA profiles of non-coding regions, including centromeric landscapes of exceptional responders or survivors and patients with poor response.

## Materials/subjects and methods

### Patient selection

**Fig. 1:**
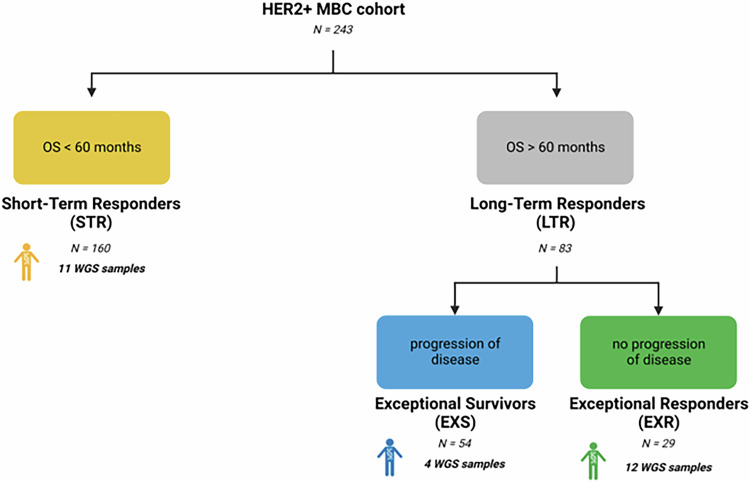
Schematic overview of the patient cohort selection and tumor sub-set strategy for whole genome sequencing. All patients diagnosed with HER2 positive MBC were categorized based on OS and/or PFS. WGS was performed on a sub-set of 11 STR, 4 ExS and 12 ExR.

A total of 243 eligible patients aged ≥18 years, diagnosed and treated for HER2-positive MBC between 2000 and 2015 were enrolled in the study. All patients received trastuzumab (single agent or combined with other treatment). Cases were selected from the “One Thousand HER2 Patients Project” database from the Department of Medical Oncology of St. Vincent’s University and St. Vincent’s Private Hospitals in Dublin, Ireland. The study protocol was approved by the St Vincent’s University Hospital Ethics & Medical Research Committee (Ref: RS19-076) and conducted in accordance with the Declaration of Helsinki. Patients were recruited with written informed consent for research whole genome analyses. Exceptional responders’ eligibility criteria were defined as an overall survival (OS) greater than 60 months with no progression of disease. Exceptional survivors (ExS) were classified as OS greater than 60 months with progression of disease. Short-term responders (STR) were patients with an OS of less than 60 months and showed disease progression (Fig. 1).

### DNA extraction

Somatic DNA was extracted from archival formalin-fixed paraffin embedded (FFPE) tumor tissues using GeneJET FFPE DNA Purification Kit (#K0881, Thermo Fisher Scientific, Waltham, MA USA) in accordance with the manufacturer’s instructions. Germline DNA was extracted from blood samples using the DNeasy Blood & Tissue Kit (#69504, Qiagen, Venlo, the Netherlands).

### DNA sequencing

Somatic DNA was extracted from 27 archival FFPE tumor tissues (20 primary tumors and 7 metastatic sites) and matching bloods. Whole-Genome Sequencing (WGS) was performed using DNBSEQ Technology (PE100 sequencing read length) on samples which passed library preparation and quality control filters at a depth of 60X for somatic samples and 30X for their matched controls (germline DNA from blood). One sample was analyzed without a match control (primary tumor only for case EXS072).

### Bioinformatics and Copy Number (CN) analysis

Reads were trimmed using sickle [16], then mapped using bwa on hg38 [17], duplicates were removed using GATK MarkDuplicates [18]. Indexing of the bam files was done by sambamba [19], evaluation of depth was performed using mosdepth [20], and evaluation of coverage was performed using samtools [21].

Copy Number Alterations (CNA) were identified using Control-FREEC [22] and then log2 normalized. Comparison of CNA profiles was performed using CNVkit [23]. RefGene database [24] and intersectBed tool [25] were used for the annotation and identification of the detected CNA. CNA burden was defined and calculated as the fraction of the genome altered by CNA. CNA status of the centromeric regions was estimated from the number of copies obtained at loci within the centromere coordinates for each chromosome (Supplemental Table S1).

### Digital PCR of chromosome 4 centromere D4Z1

The copy number of the active higher-order-repeat (HOR) D4Z1 was measured by dPCR as a measure of centromere copy number of chromosome 4. DNA samples were quantified by Nanodrop. DNA was AluI digested through 15-min incubation at 37 °C with AluI enzyme (#R0137S, New England Biolabs, Ipswich, MA USA), followed by 20-min incubation at 80 °C to inactivate the enzyme. D4Z1 copy number and reference gene copy numbers were measured by dPCR using specific primers (Supplemental Table S2). dPCR reactions were performed with 0.375-350 ng DNA using Absolute Q™ DNA Digital PCR Master Mix (#A52490, Thermo Fisher Scientific) with SYBR green (#S7567, Thermo Fisher Scientific), following the manufacturer’s instructions, and at the following cycling steps: 10 min at 96 °C, 40x (5 min at 96 °C, 15 s at 60 °C)(QuantStudio Absolute Q Digital PCR System, Thermo Fisher Scientific).

D4Z1 copy number was normalized to the reference genes RHOT1 (chr17) and MED19 (chr11) using the following formula, and the average D4Z1 CN from both normalizations was used for analysis.

$${{\rm{CN}}}_{{\rm{D}}4{\rm{Z}}1}={{\rm{C}}}_{{\rm{D}}4{\rm{Z}}1}/{{\rm{C}}}_{{\rm{ref}}}* ({{\rm{M}}}_{{\rm{D}}4{\rm{Z}}1}/{{\rm{M}}}_{{\rm{ref}}})* 2$$

With C being concentration (copies/µl) and M being mass of DNA (ng).

### Statistical analysis

Patients were divided into 3 groups depending on their treatment response: ExR, ExS and STR. Progression-free survival (PFS) was measured in months from the date since first trastuzumab treatment for metastatic disease (HER2-positive MBC) to the date of progression or last follow-up date. Overall Survival (OS) was measured in months from the date time since first trastuzumab treatment for metastatic disease to the date of death from any cause. The Kaplan-Meier method was used for the analysis of PFS and OS to compare progression or all-cause mortality dependent on treatment response, clinical characteristics, and centromere copy number (CCN) with SPSS (V30.0.0.0).

The statistical significance of the CCN status between the 3 groups of interest (ExR, ExS and STR) for each chromosome was determined using a one-way analysis of variance (ANOVA) with R (V4.2.0), corrected using the Bonferroni correction and Tukey’s HDS test to detect differences between groups (Supplementary Fig. S1). Differences of p < 0.05 were considered statistically significant.

## Results

### Patient characteristics

**Fig. 2:**
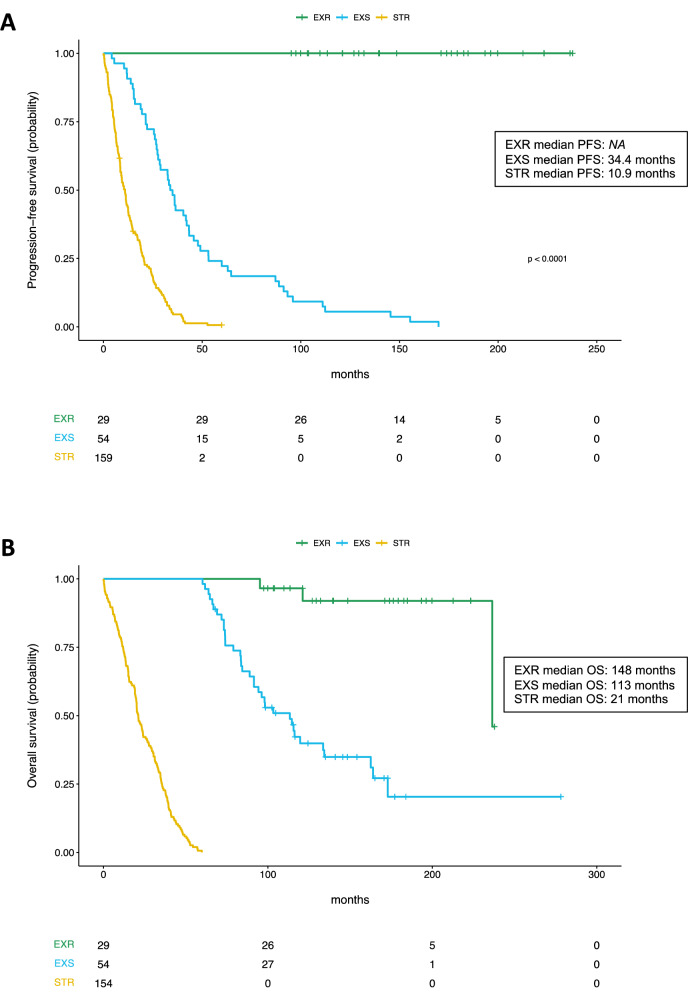
Kaplan-Meier curves of progression-free survival (PFS) and overall survival (OS) in the full cohort of ExR, ExS and STR patients. **A** Progression-free survival (PFS); **B** Overall survival (OS); time since first trastuzumab for metastatic disease in months for exceptional responders (ExR, green line), exceptional survivors (ExS, blue line) and short-term responders (STR, yellow line).

A total of 243 patients diagnosed and treated for HER2-positive MBC between 2000 and 2015 were enrolled in the study. Twenty-nine cases (12%) were identified as ExR with a median OS of 148 months (range 95.1–238 months), 54 (22%) were identified as ExS with a median OS of 113 months (range 60.2–278 months) and 160 (66%) were identified as STR with a median OS of 21 months (range 0–59 months) (Fig. 1 and Supplemental Table S3). The median progression-free survival (PFS) for the ExS and STR were 34.4 months (95% CI 27.7–43.4 months) and 10.9 months (95% CI 8.54–12.6 months) respectively (Fig. 2 and Supplemental Table S4).

All 243 HER2-positive MBC patients received trastuzumab, single agent or combined with other treatment. One hundred and fifty-three patients (63.0%) received a combination of trastuzumab with a taxane agent (including 28/29 of ExR, 39/54 of ExS and 86/160 of STR). Overall, 44 patients (18.1%) demonstrated a complete response (CR), of which 50% were ExR, 34% were ExS and 16% were STR, however all 7 STR who initially obtained CR experienced subsequent relapse within 32 months (median of 18.5 months). De novo metastases were observed in 90/243 patients (37%), of which 18/29 (62%) were ExR, 19/54 (35%) were ExS, and 53/160 (33%) were STR. Metastases were mainly located in the lung, liver, lymph nodes, brain and bone (Supplemental Table S3).

The whole genomes of 27 cases were sequenced: 26 paired samples (tumor tissue with matched control to identify genetic alterations that are unique to the tumor) and 1 tumor sample (without matched control), consisting of 12 ExR, 4 ExS and 11 STR cases. All sequenced patients were treated with trastuzumab, all ExR (100%, 12/12) and half of ExS (50%, 2/4) received trastuzumab combined with a taxane agent whereas only 27% of STR patients (3/11) did. The majority, 75% (9/12) of ExR achieved complete response (CR), 1 achieved partial response (PR) and 2 achieved stable disease (SD) (Table 1).

**Table 1:** Clinical characteristics of the whole genome sequenced cases.

	ExR	ExS	STR
	(*n* = 12)	(*n* = 4)	(*n* = 11)
**Death event**	0	4	11
**Mean PFS (months)**	*NA*	62.8	5.89
**Mean OS (months)**	158	102	11.9
***de novo*** **metastasis**			
yes	6	2	0
no	6	2	11
**Best treatment response**			
complete response (CR)	9	2	0
partial response (PR)	1	1	2
stable disease (SD)	2	1	1
other	0	0	8
**Drug category regimen**			
single agent trastuzumab	0	0	2
trastuzumab with taxane agent	12	2	3
Trastuzumab with hormone therapy	0	0	1
Trastuzumab with other chemotherapy	0	2	5
**Trastuzumab pretreated**			
yes	0	1	6
no	12	3	5

### Genome-wide CNA profile and burden

**Fig. 3:**
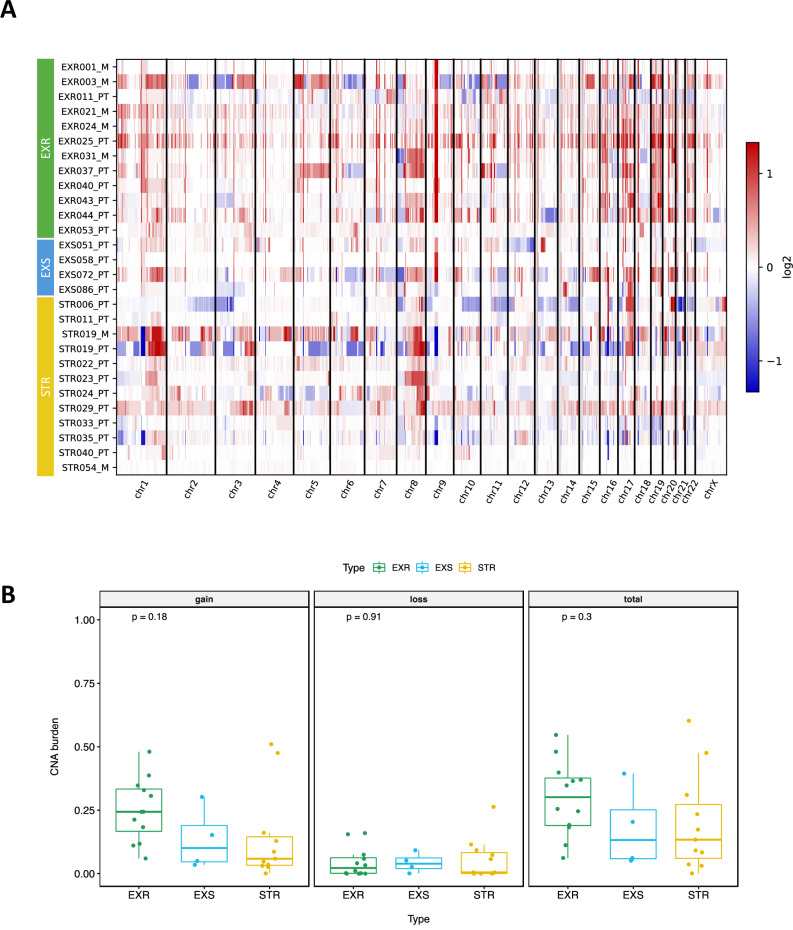
WGS genome-wide copy number alteration (CNA) landscape. **A** Heatmap of the log2 normalized CNA profiles of the whole genome of the 12 ExR, 4 ExS and 11 STR samples. Red color indicates a gain of copy; blue indicates a loss of copy and white indicates a normal copy number (2 copies). ExR samples are more impacted by gain rather than loss of copy overall; **B** Fractions of the genome altered by CNA in terms of loss, gain and both (total).

Whole genome analysis revealed that ExR samples were more impacted by global genome gain rather than loss of CNA overall (Fig. 3A) with a median of 24% ExR tumors genome amplified (range 6–48%), compared to the ExS with a median of 5% (range 3–15%) and the STR with a median of 6% amplification (range 0.1–51%) (Fig. 3B). ExS samples exhibited a trend towards greater similarity with ExR samples in terms of copy number gain; however, these observations did not reach statistical significance, likely due to the limited number of ExS samples.

#### Centromere copy number (CCN) is associated with exceptional response and overall survival

**Fig. 4:**
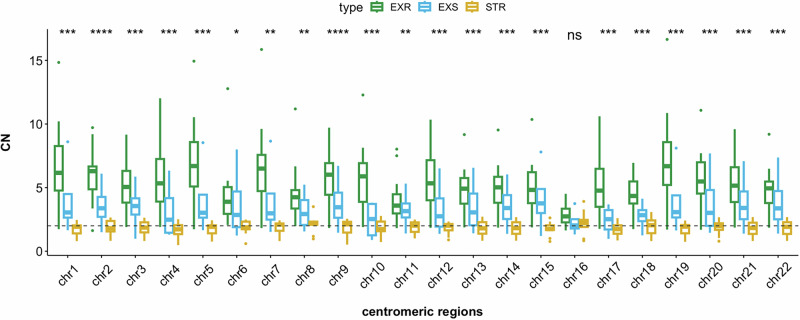
Copy number alteration (CNA) status of the centromeric region of each chromosome. A significantly higher copy number of the centromeric regions of chromosomes was detected in the ExR (green), compared to the ExS (blue) and the STR (yellow) somatic samples. Pairwise comparisons were performed using Bonferroni correction (ExR-ExS *p* = 1.8e^-14^; ExR-STR *p* = 2e^-16^ and ExS-STR *p* = 2.5e^-10^).

A higher CNA status of the centromeric regions across the genome was detected in the ExR compared to the ExS and STR samples (Fig. 4). Significant differences of the CCN status were observed in all autosomes but chromosome 16. Comparisons of the overall CCN status between the three groups, ExR-STR, ExR-ExS, and ExS-STR were significant with Bonferroni adjusted *p*-values of 2e^-16^, 1.8e^-14^, and 2.5e^-10^ respectively. Additionally, Kaplan Meier and Cox regression analysis found a significant association between CCN and OS for all autosomes (Table 2 and Supplemental Fig. S2). We also showed that the CCN amplifications in the genome are independent of the chromosome arms, and genes located near the centromeres, such as *ERBB2* (amplified in all HER2-positive MBC patients) were independent of the CCN status (Supplemental Fig. S3).

**Table 2:** Univariate Cox regression analysis of continuous CCN and overall survival.

chr	HR	95% CI	p-value
1	0.527	0.345–0.805	0.003
2	0.495	0.0328–0.746	<0.001
3	0.469	0.301–0.730	<0.001
4	0.497	0.311–0.796	0.004
5	0.535	0.353–0.810	0.003
6	0.526	0.304–0.910	0.022
7	0.55	0.359–0.842	0.006
8	0.418	0.235–0.743	0.003
9	0.486	0.317–0.746	<0.001
10	0.461	0.292–0.729	<0.001
11	0.384	0.202–0.728	0.003
12	0.457	0.275–0.759	0.002
13	0.491	0.308–0.782	0.003
14	0.478	0.302–0.759	0.002
15	0.492	0.318–0.761	0.001
16	0.493	0.244–0.996	0.049
17	0.355	0.190–0.663	0.001
18	0.427	0.254–0.718	0.001
19	0.533	0.354–0.804	0.003
20	0.522	0.345–0.790	0.002
21	0.512	0.332–0.791	0.003
22	0.51	0.328–0.793	0.003

#### Centromere copy number (CCN) as a prognostic classifier for MBC in multivariate analysis

Next, we investigated if centromere copy number (CCN) can be used to identify patients with exceptional prognosis in combination with clinical characteristics. CCN are likely to be co-linear variables for the different chromosomes as CNAs are most likely caused by the same mechanism, irrespective of the chromosome [26, 27]. Therefore, amplification above the median CCN was determined as positive. Univariate analyses of clinicopathological features revealed de novo metastasis (HR 0.210; *p* = 0.042); chemotherapy pretreatment (HR 1.001; *p* = 0.028), visceral disease (HR 4.177; *p* = 0.016), number of metastatic sites (>5) (HR 10.927; *p* = 0.045) and CCN amplification (HR 0.05; *p* < 0.0001) were significantly associated with OS. Multivariate Cox regression showed that only CCN amplification remained significant as an independent prognostic variable for OS (HR 0.041; *p* = 0.004) (Table 3).

**Table 3:** Univariate and Multivariate Cox regression analysis of clinical characteristics and centromere copy number amplification.

		Univariate analysis		Multivariate analysis	
Variable		HR (95% CI)	p-value	HR (95% CI)	*p*-value
Age, years	continuous	1.005 (0.966–1.045)	0.796		
Primary ER status	negative	reference			
	positive	0.993 (0.934–1.05)	0.831		
De novo metastasis	no	reference			
	yes	0.210 (0.046–0.942)	**0.042**	0.515 (0.791–3.362)	0.489
Chemo pretreatment	no	reference			
	yes	1.001 (1.000–1.002)	**0.028**	0.999 (0.997–1.001)	0.566
Visceral disease	no	reference			
	yes	4.177 (1.300–13.419)	**0.016**	3.561 (0.830–15.279)	0.087
Number of metastatic sites	1	reference			
	2	1.376 (0.352–5.37152)	0.645		
	>5	10.927 (1.05–113.271)	**0.045**	1.673 (0.112–24.907)	0.709
CCN amplification	no	reference			
	yes	0.050 (0.010–0.243)	**<0.0001**	0.041 (0.004–0.358)	0.004

Univariate *p* < 0.05 highlighted in bold included in Multivariate Cox regression.

#### Digital PCR assay as a proof-of-concept study to identify chromosome 4 centromere copy number (C4CCN)

**Fig. 5:**
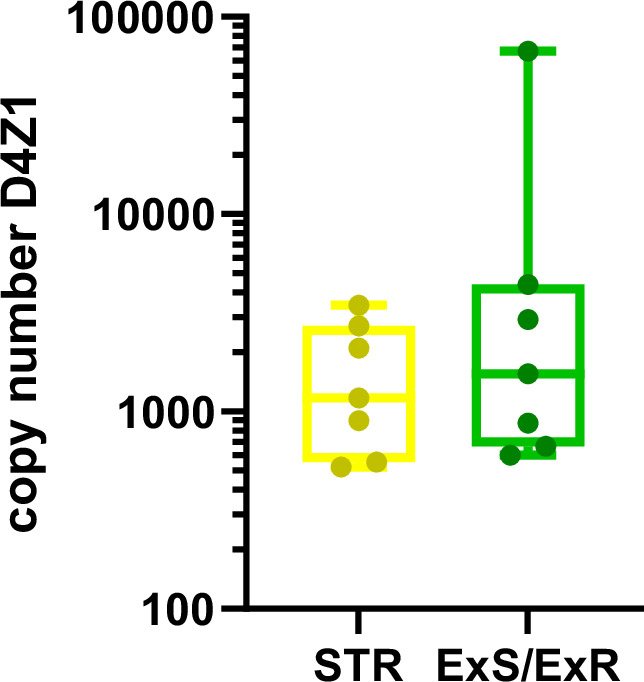
Digital PCR detection of chromosome 4 centromere copy number (C4CCN). Chromosome 4 centromere copy number (C4CCN) distribution of D4Z1 was determined by dPCR in STR and ExR/ExS patients.

Our results suggest that CCN status could be a powerful biomarker to predict MBC patient response. However, standard whole genome sequencing is a relatively expensive and time-consuming technique which is not optimized for the analysis of centromeric regions. We therefore sought to investigate the predictive potential of CCN analysis by digital PCR. We targeted D4Z1, the active higher-order repeat of chromosome 4, using a previously published primer set [28] to investigate C4CCN in 7 STR and 7 ExR/ExS. The median number of D4Z1 repeats in STR patients was 1170.80 (range 523.73-3459.90) and for ExR/ExS patients was 1557.74 (range 602.49-66885.45) (Fig. 5). The samples were dichotomized based on the overall median C4CCN (1364.27) and plotted as Kaplan-Meier survival curves (Supplemental Fig. S4 and Supplemental Table S5). No significant association was found between C4CCN and OS (*p* = 0.872) or PFS (*p* = 0.302). Similarly, no significant association was found between continuous C4CCN and OS or PFS by Cox regression analysis (OS: HR 1.004, *p* = 0.815; PFS: HR 1.000, *p* = 0.463).

## Discussion

Copy number alteration (CNA) burden can be used as a prognostic factor for recurrence and/or overall survival in multiple cancer types [29, 30]. CNA burden refers to the fraction of the genome altered, involving duplications and deletions of large DNA fragments. This type of genetic aberration can affect coding regions of the genome, leading to the over or under expression of oncogenes or tumor suppressors. However, non-coding regions such as centromeres, due to their highly repetitive structures, pose challenges for accurate CNA profiling [31]. Centromeres are epigenetically defined as the chromosomal region where histone H3 variant centromeric protein A (CENP-A) binds and the kinetochore forms. In humans, centromeres span multiple megabases and are characterized by highly repetitive sequences [32, 33]. The active core of the centromere is composed of alpha satellite repeat arrays: a collection of highly similar ~171 bp monomers that are organized in higher-order repeats (HORs). The active HOR is flanked by shorter, more divergent HORs and other repeat structures at the pericentromeres [32].

Here, we investigated WGS data of 27 HER2-positive MBC patients and observed a significant difference in CNA between the ExR, ExS and STR at the centromeric region of the chromosomes. Amplification of the centromeric regions in one patient were detected in germline and tumoral DNA. Our results suggest that gains or amplifications of these regions, rather than a copy number gain of the whole chromosomes, stratified patients according to their treatment response. We found that ExR tumors consistently exhibited a gain of at least one additional copy of the centromeric regions, whereas STR tumors predominantly maintained a normal copy number or showed a loss of one copy in these regions. These observations suggest that patients with tumors that harbor centromeric region amplifications benefit more from treatment with a combination of trastuzumab with a taxane agent.

Due to the extensive variability of centromeric sequences in the population, it remains unclear whether centromeric size or copy number alterations directly influence centromere activity. The expansion and contraction of centromere satellite arrays have previously been shown to either involve unequal crossover [34] and gene conversion during homologous recombination to repair DNA double strand breaks (DSB) [35], or due to break-induced replication (BIR) where hundreds of kilobases are replicated by the one-sided DSB repair mechanism [36]. Showman et al. [27] observed centromere expansion is favored over contraction during cancer cell line clonal evolution. Zhang et al. [37] developed a centromere and kinetochore gene expression score (CES) whereby the overexpression of 14 centromere and kinetochore protein genes correlated with increased levels of CNA and mutations. A high CES signature predicted outcomes in breast and lung cancers receiving chemotherapy or radiotherapy. Additionally, cell lines with high CES demonstrated increased sensitivity to topoisomerase I inhibitors. Furthermore, response to therapy has been previously shown to be modulated by centromere amplifications with several studies highlighting survival benefit to patients exhibiting chromosome 17 centromere CEP17 duplications treated with anthracyclines [38–40]. However, the role of centromere amplification in exceptional response to taxane and trastuzumab is not fully clear. Sun et al. demonstrated that elevated expression of CENP-A, which maintains the structure, formation, number and function of centromeres, can be used as a prognostic and predictive cancer biomarker as its expression strongly correlates with a positive response to taxane-based regimens in breast cancer [41]. CENP-A overexpression can lead to the formation of ectopic neocentromeres and kinetochores in chromosome arms contributing to genome instability [42]. As CENP-A is located on the centromeres, centromere amplification may affect CENP-A expression. Lahaye et al. observed that viral proteins can cause amplification of centromere DNA and nuclear cGAS amplification uncovering a non-mitotic, immune-activating role of centromeres [43]. Indeed, activation of a cGAS-STING-mediated immune response has been shown to predict response to neoadjuvant chemotherapy in early breast cancer [44], and conversely the suppression of the cGAS-STING pathway is associated with trastuzumab resistant breast cancer [45].

We analyzed the correlation between individual CCN and OS and found a significant association for each autosome between amplification and OS. Additionally, multivariate Cox regression showed that the presence of CCN amplification alone remained an independent predictor of overall survival.

Previous studies have shown innovative approaches such as digital PCR that can allow a more precise investigation of the high-order repeats within centromeric regions [31]. To further explore the precise centromere region amplification, we investigated whether CNA analysis of the centromere on chromosome 4 by dPCR could be used as a biomarker for OS in a small proof-of-concept experiment. We used a primer targeting a monomer in the active HOR of chromosome 4 (D4Z1) which has been used previously to study human centromere variation [28]. The aHOR is split over three regions (chr4:49705155-50433557, 52115487-54870510, 54980291-55199795) and spans 3.49 Mb in the T2T-CHM13 genome reference. We observed a > 100-fold variation in the number of repeats detected across our sample cohort with numerically increased repeats in ExR/ExS patient tumors compared to STR but we did not observe a significant difference in CCN or an association with OS. This study has several limitations as WGS is not optimal for investigation of centromeric regions as the combination of short read length and repetitiveness of centromeric sequences as well as high homology between several chromosomes may hinder accurate read alignment. While this is unlikely to affect the association of centromere amplification with OS, as amplification was observed across all centromeres, it is likely that this analysis will have missed smaller variations, which would require inclusion of telomere-to-telomere long read sequencing as well as fine scale mapping of a multi centromere panel of dPCR primers. In addition, our results are preliminary due to the restricted sample size and would require confirmation in larger prospective studies specifically designed to validate the predictive role of centromeric amplification. Although our previous study performed WES in MBC patients and observed non-significant numerically lower CNA burden in ExR compared to non-responders in the gene coding regions [12]. Review of the coding region from our current WGS study also observed a non-significant difference between the cohorts, however ExR contained a numerically higher CNA compared to STR (Supplemental Fig. S5).

In conclusion, our results suggest that the centromere amplification may serve as a promising biomarker for identifying exceptional responses in HER2-positive MBC patients. Enhanced insights into centromeric dynamics could better refine patient stratification, enabling the selection of patients most likely to benefit from the combination of trastuzumab with taxanes, supporting a more personalized treatment strategy.

## Supplementary information

Supplemental material

## Data Availability

The datasets generated during and/or analysed during the current study are available in the European Genome Archive repository, under study ID EGA00001719456.
