# Supplementary material for: Chromosome centromere copy number amplification associated with exceptional response in HER2-positive metastatic breast cancer patients
Source: Oncogene. 2025 Dec 20;45(4):549–57. doi: 10.1038/s41388-025-03667-8 (PMC12815647; doi:10.1038/s41388-025-03667-8)
Supplement: Supplementary file 1 — Supplemental material [file 41388_2025_3667_MOESM1_ESM.docx]

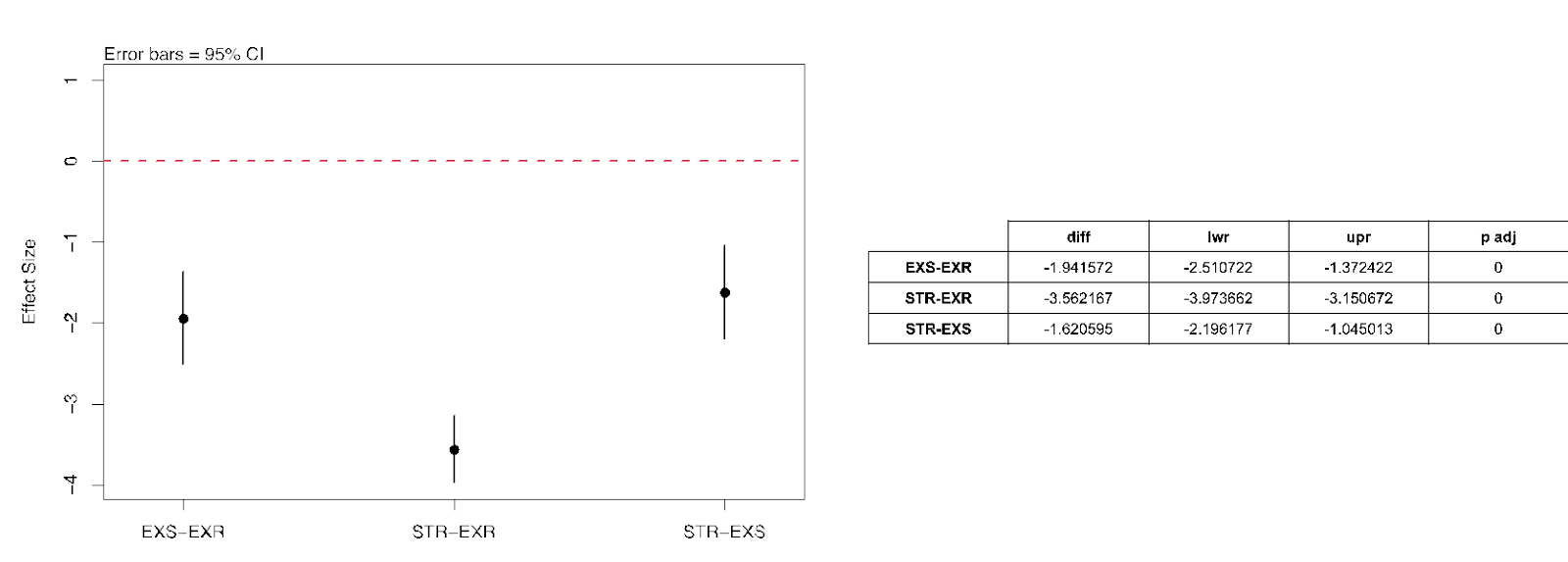


***Figure S1. Tukey comparisons of centromeric CN means between ExR, ExS and STR.***

*Post hoc ANOVA test (Tukey) performed using R (V4.2.0) and R packages rstatix, AICcmodavg, ggplot2, ggpubr, tidyverse, broom and multcompView.*

*
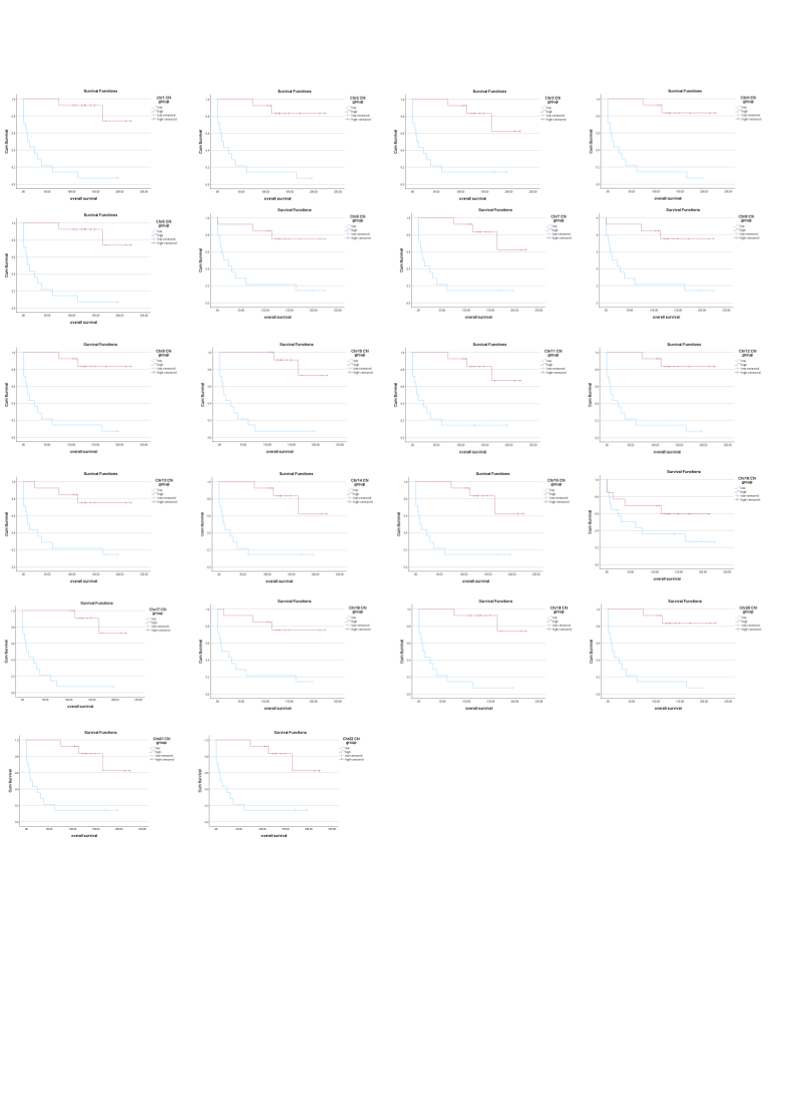
*

***Figure S2. Kaplan Meier curves of overall survival in ExR, ExS, and STR patients divided by centromeric copy number.*** *Patients were divided into a low or high CCN group based on the median CCN.*


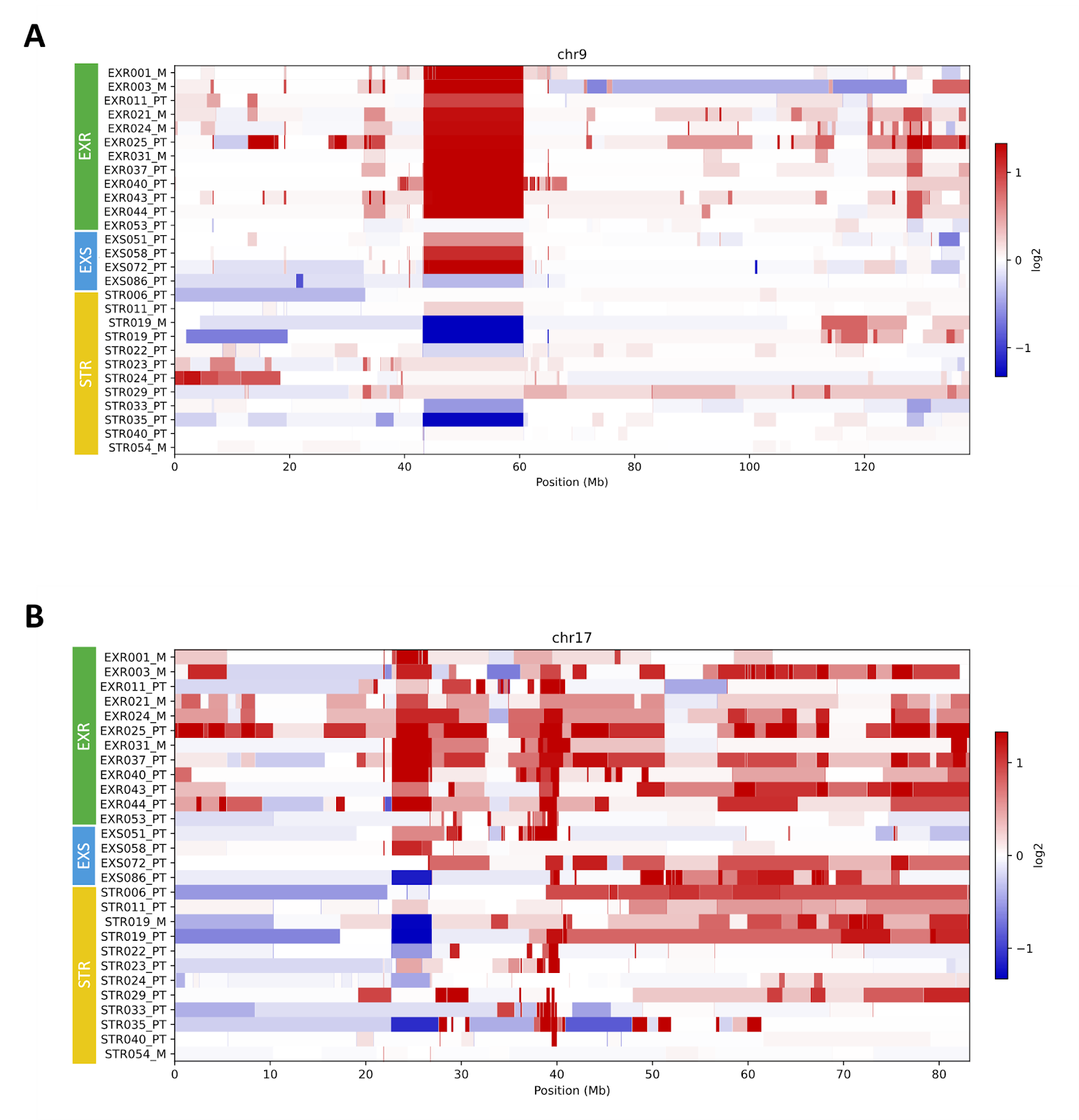


***Figure S3. Copy number aberration profiles of chromosomes 17.***

*Heatmap of the log2 normalized CNA profiles of the whole genome of the 12 EXR, 4 EXS and 11 STR samples. Red color indicates a gain of copy, blue indicates a loss of copy and white indicates a normal copy number (2 copies)*

*
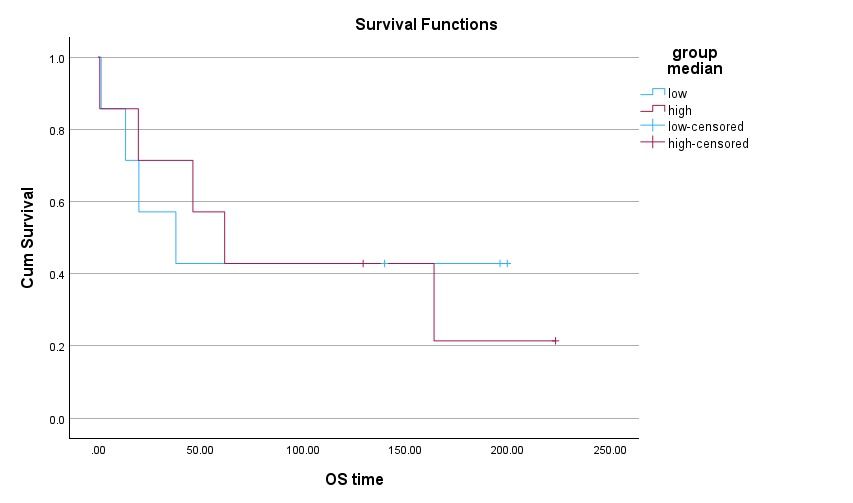
*

*
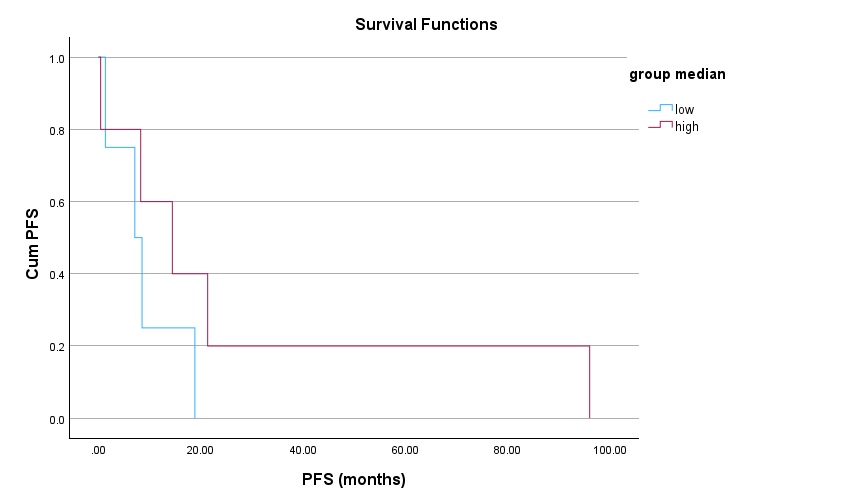
*

**Figure S4. *Kaplan Meier curves of overall survival and progression-free survival in ExR, ExS, and STR patients grouped by D4Z1 copy number.***

*
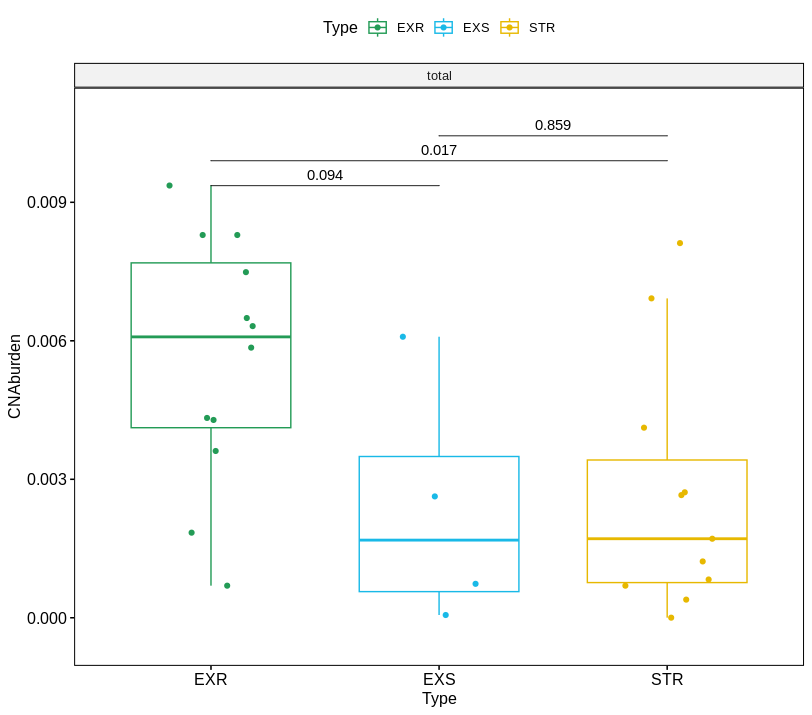
*

***Figure S5. CNA burden of the coding regions.****Fractions of the genome altered by CNA in terms of loss and gain (total) for the EXR, EXS and STR groups.*

***Table S1. Coordinates of the selected centromeric regions (hg38).***

*Coordinates used to estimate the copy-number status of the centromeric regions.*

| ***chromosome*** | ***start*** | ***end*** |
| --- | --- | --- |
| chr1 | 122500000 | 124000000 |
| chr2 | 93000000 | 94000000 |
| chr3 | 91551000 | 93400000 |
| chr4 | 49650000 | 49700000 |
| chr5 | 47251000 | 48600000 |
| chr6 | 58451000 | 58600000 |
| chr7 | 59851000 | 60000000 |
| chr8 | 44100000 | 44500000 |
| chr9 | 44900000 | 45400000 |
| chr10 | 39700000 | 39900000 |
| chr11 | 53951000 | 54300000 |
| chr12 | 35400000 | 37200000 |
| chr13 | 17500000 | 18000000 |
| chr14 | 17651000 | 18200000 |
| chr15 | 18400000 | 19700000 |
| chr16 | 36110000 | 36210000 |
| chr17 | 25950000 | 26500000 |
| chr18 | 20610000 | 20800000 |
| chr19 | 26300000 | 26900000 |
| chr20 | 26551000 | 28000000 |
| chr21 | 12310000 | 12400000 |
| chr22 | 14500000 | 15000000 |

***Table S2. dPCR primers***

| ***Gene*** | ***primer*** | ***Assay ID*** |
| --- | --- | --- |
| *D4Z1* | *FW 5’ CTG TAG TAT CTG GAA GTG GAC ATT 3’* | *ThermoFisher* |
|  | *RV 5’ GGT TCA ACT GTG TTC GTT TAG G 3’* |  |
| *RHOT1* | *FW 5’ CGT AGC TGC AAA GTC AGA CC 3’* | *Hs.PT.58.40542312.g, Integrated DNA technologies, Coralville, IA USA* |
|  | *RV 5’ GTC AAT TTA ACA AAG AA TCC TTA CTG G 3’* |  |
| *MED19* | *FW 5 GAT TGA TCT GCC TGG TTC CC 3’* | *Hs.PT.58.26606431.g, Integrated DNA technologies, Coralville, IA USA* |
|  | *RV 5’ CAG TGT GGA GGC GGA AG 3’* |  |

***Supplemental Table S3. Clinical characteristics of the HER2-positive MBC cohort.***

|  | ***ExR*** | ***ExS*** | ***STR*** |
| --- | --- | --- | --- |
|  | *(n=29)* | *(n=54)* | *(n=160)* |
| ***Median age (range) at first anti-HER2 line, years*** | *50 (range 30-77)* | *54 (range 25-79)* | *63 (range 26-88)* |
| ***Median survival, months*** |  |  |  |
| ***OS*** | *148 (range 95-238)* | *113 (range 60.2-278)* | *21 (<60)* |
| ***PFS*** | *NA* | *34.4 (4-170)* | *10.9 (<60)* |
| ***de novo metastasis*** |  |  |  |
| ***yes*** | *18 (62%)* | *19 (35%)* | *53 (33.1%)* |
| ***no*** | *11 (38%)* | *35 (65%)* | *103 (64.4%)* |
| ***unknown*** | *0 (0%)* | *0 (0%)* | *4 (2.5%)* |
| ***ER/PR status (primary tumor or metastases)*** |  |  |  |
| ***positive*** | *15 (51.7%)* | *23 (43%)* | *55 (34.375%)* |
| ***negative*** | *12 (41.3%)* | *25 (45%)* | *76 (47.5%)* |
| ***unknown*** | *2 (7.0%)* | *6 (12%)* | *29 (18.125%)* |
| ***Number of metastatic sites*** |  |  |  |
| ***1*** | *21 (71.0%)* | *25 (46.1%)* | *53 (33.1%)* |
| ***2*** | *4 (16.1%)* | *20 (38.5%)* | *68 (42.5%)* |
| ***3 or more*** | *4 (12.9%)* | *9 (15.4%)* | *36 (22.5%)* |
| ***unknown*** | *0 (0%)* | *0 (0%)* | *3 (1.9%)* |
| ***Location of metastases*** |  |  |  |
| ***lung*** | *5* | *13* | *54* |
| ***lymph nodes*** | *16* | *24* | *59* |
| ***brain*** | *0* | *3* | *18* |
| ***liver*** | *8* | *15* | *67* |
| ***bone*** | *7* | *29* | *91* |
| ***chest wall recurrence*** | *2* | *3* | *10* |
| ***Best treatment response*** |  |  |  |
| ***complete response (CR)*** | *22 (75.9%)* | *15 (27.8%)* | *7 (4.4%)* |
| ***partial response (PR)*** | *4 (13.8%)* | *29 (53.7%)* | *54 (33.8%)* |
| ***stable disease (SD)*** | *2 (6.9%)* | *7 (13.0%)* | *37 (23.1%)* |
| ***progression of disease (POD)*** | *0 (0%)* | *2 (3.7%)* | *41 (25.6%)* |
| ***unknown*** | *1 (3.4%)* | *1 (1.8%)* | *21 (13.1%)* |
| ***Drug category regimen*** |  |  |  |
| ***single agent trastuzumab*** | *0 (0%)* | *1 (1.8%)* | *18 (11.25%)* |
| ***trastuzumab with chemotherapy*** | *28 (96.6%)* | *46 (85.2%)* | *115 (71.875%)* |
| ***trastuzumab with hormonal therapy*** | *0 (0%)* | *4 (7.4%)* | *13 (8.125%)* |
| ***unknown*** | *1 (3.4%)* | *3 (5.6%)* | *14 (8.75%)* |

***Table S4. PFS and OS statistics.*** *Kaplan Meier survival analysis performed using R (V4.2.0) and R packages survival and survminer.*

|  | **EXR** | **EXS** | **STR** |
| --- | --- | --- | --- |
|  | (n=29) | (n=54) | (n=160) |
| **Progression-Free Survival** |  |  |  |
| records | 29 | 54 | 159 |
| events | 0 | 54 | 156 |
| mean, months | *NA* | 46.5 | 15.1 |
| median, months | *NA* | 34.4 | 10.9 |
| 0.95 LCL, months | *NA* | 27.7 | 8.54 |
| 0.95 UCL, months | *NA* | 43.4 | 12.7 |
| **Overall Survival** |  |  |  |
| records | 29 | 54 | 159 |
| events | 3 | 36 | 154 |
| mean, months | 157 | 139 | 23.8 |
| median, months | 148 | 113 | 21.1 |
| 0.95 LCL, months | *NA* | 91.5 | 19.3 |
| 0.95 UCL, months | *NA* | 152 | 26.4 |

***Table S5. D4Z1 copy number as measured by dPCR and associated PFS and OS.***

| **ID** | **D4Z1_avg** | **OS** | **PFS** | **WGS** |
| --- | --- | --- | --- | --- |
| EXR004 | 665.88 | 199.69 | NA | N |
| EXR021 | 602.49 | 139.79 | NA | Y |
| EXR024 | 1557.74 | 129.31 | NA | Y |
| EXR043 | 4405.96 | 223.28 | NA | Y |
| EXS051 | 2917.61 | 163.94 | 95.97 | Y |
| EXR053 | 874.31 | 196.17 | NA | Y |
| EXS085 | 66885.45 | 61.7 | 21.36 | N |
| STR005 | 899.56 | 13.31 | 7.13 | N |
| STR023 | 2711.97 | 0.72 | 0.46 | Y |
| STR024 | 1170.8 | 1.38 | 1.38 | Y |
| STR049 | 3459.9 | 19.55 | 8.28 | N |
| STR052 | 555.08 | 19.81 | 8.54 | N |
| STR053 | 2092.07 | 46.19 | 14.46 | N |
| STR054 | 523.73 | 37.88 | 18.86 | Y |
